# MRI-Based Radiomics in Bladder Cancer: A Systematic Review and Radiomics Quality Score Assessment

**DOI:** 10.3390/diagnostics13132300

**Published:** 2023-07-06

**Authors:** Bianca Boca, Cosmin Caraiani, Teodora Telecan, Roxana Pintican, Andrei Lebovici, Iulia Andras, Nicolae Crisan, Alexandru Pavel, Laura Diosan, Zoltan Balint, Monica Lupsor-Platon, Mircea Marian Buruian

**Affiliations:** 1 Department of Radiology, “George Emil Palade”, University of Medicine, Pharmacy, Science and Technology of Targu Mures, 540139 Targu Mures, Romania; petresc.bianca@elearn.umfcluj.ro (B.B.); alexandru.pavel@elearn.umfcluj.ro (A.P.); mircea.buruian@umfst.ro (M.M.B.); 2 Department of Medical Imaging and Nuclear Medicine, “Iuliu Hațieganu” University of Medicine and Pharmacy Cluj-Napoca, 400012 Cluj-Napoca, Romania; monica.lupsor@umfcluj.ro; 3 Department of Urology, “Iuliu Hațieganu” University of Medicine and Pharmacy Cluj-Napoca, 400012 Cluj-Napoca, Romania; dr.iuliaandras@gmail.com (I.A.); drnicolaecrisan@gmail.com (N.C.); 4 Department of Urology, Clinical Municipal Hospital, 400139 Cluj-Napoca, Romania; 5 Department of Radiology, “Iuliu Hațieganu” University of Medicine and Pharmacy Cluj-Napoca, 400012 Cluj-Napoca, Romania; roxana.pintican@gmail.com (R.P.); andrei1079@yahoo.com (A.L.); 6 Department of Computer Science, Faculty of Mathematics and Computer Science, “Babes-Bolyai” University, 400157 Cluj-Napoca, Romania; laura.diosan@ubbcluj.ro; 7 Department of Biomedical Physics, Faculty of Physics, “Babes-Bolyai” University, 400084 Cluj-Napoca, Romania; zoltan.balint@ubbcluj.ro; 8 Department of Radiology, Regional Institute of Gastroenterology and Hepatology “Prof. Dr. Octavian Fodor”, 400162 Cluj-Napoca, Romania

**Keywords:** bladder cancer, MRI, radiomics, radiomics quality score

## Abstract

(1): Background: With the recent introduction of vesical imaging reporting and data system (VI-RADS), magnetic resonance imaging (MRI) has become the main imaging method used for the preoperative local staging of bladder cancer (BCa). However, the VI-RADS score is subject to interobserver variability and cannot provide information about tumor cellularity. These limitations may be overcome by using a quantitative approach, such as the new emerging domain of radiomics. (2) Aim: To systematically review published studies on the use of MRI-based radiomics in bladder cancer. (3) Materials and Methods: We performed literature research using the PubMed MEDLINE, Scopus, and Web of Science databases using PRISMA principles. A total of 1092 papers that addressed the use of radiomics for BC staging, grading, and treatment response were retrieved using the keywords “bladder cancer”, “magnetic resonance imaging”, “radiomics”, and “textural analysis”. (4) Results: 26 papers met the eligibility criteria and were included in the final review. The principal applications of radiomics were preoperative tumor staging (*n* = 13), preoperative prediction of tumor grade or molecular correlates (*n* = 9), and prediction of prognosis/response to neoadjuvant therapy (*n* = 4). Most of the developed radiomics models included second-order features mainly derived from filtered images. These models were validated in 16 studies. The average radiomics quality score was 11.7, ranging between 8.33% and 52.77%. (5) Conclusions: MRI-based radiomics holds promise as a quantitative imaging biomarker of BCa characterization and prognosis. However, there is still need for improving the standardization of image preprocessing, feature extraction, and external validation before applying radiomics models in the clinical setting.

## 1. Introduction

Bladder cancer (BCa) is the second most common urological malignancy, registering over 500,000 newly diagnosed cases worldwide yearly [1]. In terms of treatment strategies, prognosis, and survival rates, the assessment of the tumoral extension into the muscular layer is crucial. According to the current guidelines, in order to differentiate between non-muscle invasive (NMIBC) and muscle-invasive (MIBC) BCa, as well as to assess its differentiation and aggressiveness, transurethral resection of the bladder tumor (TURBT) must be performed with subsequent pathological evaluation of the retrieved specimen [2]. However, this is an invasive procedure, harboring risks such as hematuria, urinary tract infection, and bladder perforation [3]. 

Preoperative imaging methods have been improved over the past decade, multiparametric magnetic resonance imaging (mpMRI) of the bladder playing a central role regarding the diagnosis and staging of BCa, accurately identifying muscular invasion in up to 85% of examinations [4]. The assessment of the muscular invasion was standardized in 2018 by Panebianco et al. [5] through the vesical imaging-reporting and data system (VI-RADS) score. Although it has reduced the interobserver disagreement regarding BCa staging, the VI-RADS score was proven to have a steep learning curve, novice radiologists requiring 150 cases before obtaining independence in terms of accurate diagnosis [6]. 

In response to these challenges, Lambin et al. [7] developed the concept of radiomics, defined as the domain that quantifies the heterogeneity of medical images, by extracting features that may not be visible to the naked eye, such as pixels’ intensity, spatial interrelationships, and derived textures. Its greatest applicability has been found in radio-oncology, as tumoral tissues are highly heterogenic structures, forming disease-specific textural patterns that are directly correlated to their histologic phenotype, thus having the potential to be used as non-invasive biopsy surrogates. In terms of imaging modalities, MRI presents the highest resolution when characterizing soft tissues, being one of the preferred imaging supports for texture analysis [8]. Recently, radiomic features have been integrated into the BCa diagnosis workflow by offering preoperative staging and tumoral grading predictions based on bladder mpMRI acquisitions. It has been shown that the addition of textural features increased the specificity of BCa diagnosis by 12% and reduced the lymph nodes understaging rate by 36% when compared to the radiologist’s mpMRI interpretation alone [9].

The aim of this paper is to perform a systematic review of the use of MR imaging radiomics for BCa staging, grading, and disease prognosis and also to determine the quality of published papers using a radiomics quality score.

## 2. Materials and Methods

### 2.1. Literature Research and Study Selection

For this systematic review, we performed a structured search of publications investigating MRI-based radiomics applications to bladder cancer according to the PRISMA (preferred reporting items for a systematic review and meta-analysis) guidelines. The following key terms were used: “radiomics” OR “texture analysis” OR “radiomic features” OR “textural features” AND “bladder cancer” OR “bladder tumor” AND “magnetic resonance imaging” OR “MRI”. Two researchers independently conducted the literature search on three electronic databases: PubMed MEDLINE, Scopus, and Web of Science, screening potential articles published before 31 March 2023. Study selection was conducted by screening the title and abstract and then retrieving the full text. Reference lists of retrieved articles were also analyzed in order to identify additional eligible papers. The list of records was screened for duplicates and, if present, they were removed. Any discrepancy between the two researchers was solved by consensus. 

Based on the following inclusion criteria, we selected the publications that:

1. evaluated BCa using an MRI-based radiomics approach.
2. provided information related to tumor characterization (grading, staging, and muscular invasion status)
3. provided information related to tumor prognosis (survival, recurrence rate, and response to neoadjuvant therapy)
4. were written in English.

Exclusion criteria included the following:

1. studies based on other imaging modalities, such as ultrasound, CT, PET-CT
2. publications designed as letters to the editor, editorial, conference abstract, review, systematic review, meta-analyses, or case reports.
3. articles focusing on methodological aspects of radiomics and artificial intelligence, without well-established clinical application
4. studies considering only semantic imaging features.
5. topics out of the purpose of this review.
6. studies with a sample size under 30.

### 2.2. Data Extraction

We used a pre-defined table to extract the following data from each selected article:

1. general features, including the name of authors, country, publication year, and journal.
2. study characteristics, including general aim, study design (prospective, retrospective), MRI technical data (e.g., type of scanner, field of strength, sequences used for radiomics analysis), sample size.
3. Details of radiomics analysis, including software used for segmentation and feature extraction, segmentation method, imaging preprocessing, number and type of extracted features, feature selection methods/machine learning classifiers, number of selected radiomics features.
4. performance or prognostic metrics of a radiomics model in terms of area under the curve (AUC) or concordance index (C-index).

Studies were divided into three groups according to their main goal: (1) predicting the grade of BCa and molecular correlates, (2) predicting the tumor stage, including muscular invasion status and lymph node status, (3) predicting the prognosis of BCa. 

### 2.3. Quality Assessement

In order to assess methodological quality regarding the radiomic workflow, the enrolled studies were evaluated using the radiomics quality score (RQS) proposed by Lambin et al. [10]. This score is a radiomic-specific quality assessment which consists of 16 criteria regarding robustness and reproducibility. Each criterion is assigned a different maximum score corresponding to its importance, and the total RQS ranges from −8 to +36 points. The score is converted into a percentage value, with 36 points corresponding to 100%. Two readers (B.B. and R.P.) independently scored the articles for each category. If disagreement occurred, a final decision was made through consensus.

A third reader (T.T.) assessed the methodological quality of each included paper regarding the risk of bias and their applicability by using the revised quality assessment of diagnostic accuracy studies (QUADAS-2) tool [11]. QUADAS-2 evaluates the risk of bias in four domains: patient selection, index test, reference standard, and flow and timing.

## 3. Results

### 3.1. Characteristics of Included Studies

**Figure 1:**
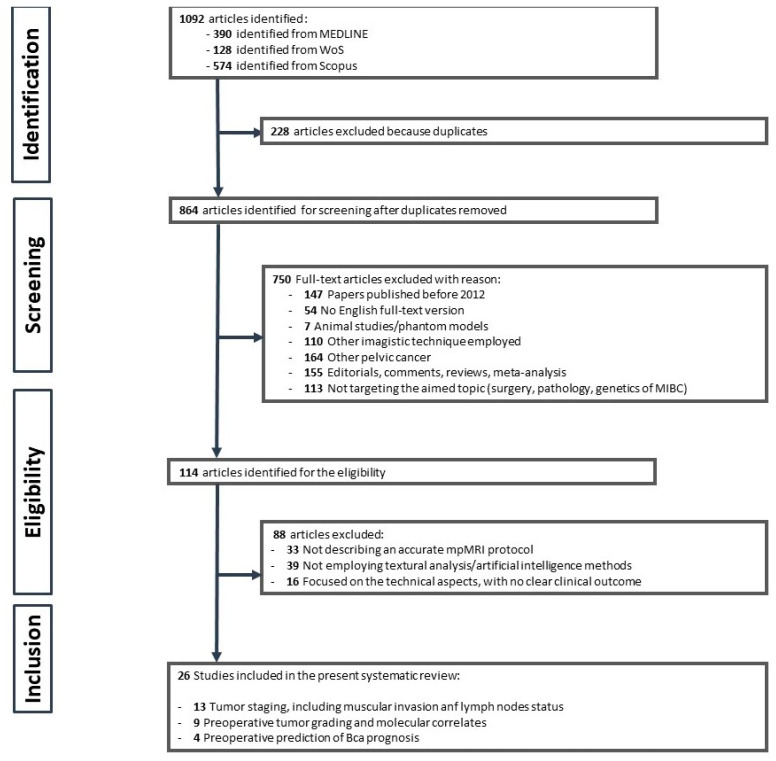
PRISMA flowchart of the screened and included studies.

A total of 1092 studies were initially identified through the literature search. After removing duplicates and screening for eligibility, 26 studies were finally included in the analysis [12,13,14,15,16,17,18,19,20,21,22,23,24,25,26,27,28,29,30,31,32,33,34,35,36,37]. Figure 1 depicts the PRISMA flowchart. The publication dates of the selected papers ranged between 2017 and March 2023, with 53% of them (14/26) being released within the last two years.

All of the included studies had a retrospective design, including 2991 patients in total. The cohort sizes ranged from 36 to 218 patients. Two thirds of the studies divided their population into a training and a test cohort, while only three of them further validated their model using an independent external validation set. 

Regarding the MRI sequences used for the extraction of radiomics features, 8 studies used only one sequence (morphological T2-weighted images [T2WI], diffusion-weighted sequence [DWI], or apparent diffusion coefficient [ADC] map) [12,14,15,21,23,25,32,36], while the rest applied a multiparametric analysis, using two or three image inputs for the development of the radiomic models. Most articles (*n* = 23) provided a description of MRI protocols, with most of the images being acquired using 3T MRI scanners. In 23 studies, at least two experienced radiologists were involved in the diagnostic and segmentation process. 

As for the segmentation strategy, in all studies except for two, the tumor delineation was performed manually, and in 20 investigations, the authors chose a volumetric approach by segmenting a volume of interest (VOI). Approximately one half of studies (*n* = 12) conducted segmentation using the freely available segmentation software ITK-SNAP (versions 3.4.0, 3.6.0 and 3.8.0), while for the feature extraction, 13 studies used the PyRadiomics package. 

The number of extracted radiomics features ranged from 36 to 15,384. The study that included the least number of features analyzed only histogram features from original and filtered images [17]. The rest of the papers extracted similar features such as shape-based features, first-order statistics, and texture features, with a mean of 1316 extracted features. 

All but three studies developed a radiomic model to predict the diagnostic or prognostic outcome. In order to select the most useful radiomics features and to reduce the effect of overfitting, various approaches to dimensionality reduction were applied. Least absolute shrinkage and selection operator (LASSO) regression was used as a machine-learning (ML) classifier in more than one half of studies (*n* = 15), followed by support vector machines (SVMs) with or without recursive feature elimination (RFE) in 13 studies. Nine investigations (34%) compared at least two ML algorithms and selected the best-performing model. The number of features included in the radiomics models varied between 3 and 157, with a mean of 22.6. 16 studies extracted only quantitative radiomics features, whereas 10 studies included both radiomic and semantic features in a combined prediction model. Four studies used data augmentation techniques such as the synthetic minority over-sampling technique (SMOTE) to rebalance data sets. 

The data from the papers are summarized in Table 1 and Supplementary Tables S1–S3.

### 3.2. Assessment of Study Quality

Table 2 presents the score of each item and the total score for each study. The mean RQS of all studies evaluated by two raters was 11.7 (32.5%) points, ranging from 3 (8.3%) to 19 (52.7%) points. Only four studies scored equal to or above 50% [21,22,28,35].

Table 3 shows the results of QUADAS analysis.

### 3.3. Prediction of BCa Grade and Molecular Correlates

Of the 26 eligible studies, 6 had the preoperative prediction of BCa pathological grade as their main objective, all of them dividing the data into low-grade and high-grade tumors [13,18,26,27,30,35]. 

All six studies reported AUCs higher than 0.85, with four of them [18,27,30,35] confirming their Rad-Score in a validation group with AUC values higher than 0.9. The best performance was achieved by Zheng et al. [27], who reported an AUC of 0.961 in the training test and 0.952 in the validation set. Their radiomic model consisted of 26 relevant features extracted from T2-WI and DCE images (late phase) and selected using the LASSO algorithm. This paper used a data augmentation technique to balance the data sets. Even after removing the synthetic sample, the AUCs of their radiomic signature were relatively similar, with values of 0.935 and 0.950 in the training and validation sets, respectively. 

Two papers built a combined model for the prediction of bladder cancer grade, all of them using a separate data set for validation [27,35]. Also in this case, the model developed in the paper of Zheng et al. [27] performed the best, yielding AUCs of 0.956 and 0.958 in the training and validation sets, respectively. For this model, the radiomics score was combined with the VI-RADS score assessed by two experienced radiologists in consensus. 

In a single study by Razik et al. [26], only first-order features were extracted, and the authors did not develop a radiomic model. They evaluated the diagnostic performance of each of the 36 extracted features and reported the best AUC of 0.897 for the following features: mean and mean of positive pixels (MPP), both extracted from ADC maps.

Three studies used radiomics features to preoperatively identify genetic and immunologic markers of BCa, including Ki-67, CD-8A, and a constructed immune prognostic signature (IPS) consisting of five immune-related genes [29,34,37]. For the prediction of Ki-67, the data set was divided into a high Ki-67 expression group (>15% cells stained) and a low Ki-67 expression group (≤15%). The built radiomics signature achieved the best performance in SMOTE-LASSO training and validation sets, with AUCs of 0.859 and 0.819, respectively [29]. The reported AUCs in the original, unbalanced data sets were comparable with SMOTE-LASSO: 0.826 and 0.793. In the paper by Zheng et al. [34], the authors demonstrated the potential of radiomics features to preoperatively predict the expression of CD8A in both the training (AUC = 0.857) and validation sets (AUC: 0.844). Liu et al. [37] developed a radiomic model which efficiently predicted the five gene-based IPS in both the training (AUC = 0.839) and validation sets (AUC = 0.819). All of these three papers developed Rad-Score using features derived from T2WI and DCE images and used LASSO algorithm as an ML classifier. None of these papers investigated the potential of complex radiological–clinical models.

### 3.4. Prediction of BCa Stage, including Muscle Invasion and N Stage

Half of the studies (*n* = 13) evaluated radiomics as a tool for the prediction of BCa stage, mainly focusing on the differentiation between non-muscle-invasive tumors (T1 stage) and muscle-invasive ones (T2 stage) [12,14,16,17,19,21,22,24,26,28,31,32,36]. All of them used the pT stage as reference standard with variations of the surgical techniques between TURBT, partial (PC), or radical cystectomy (RC).

Most of them constructed Rad-Score encompassing multiple features (between 6 and 157, whereas only 7 validated their signatures on a different data set [19,21,22,28,31,32,36]. 

Two studies, Razik et al. [26] and Lim et al. [17], analyzed only the role of first-order features for the prediction of tumor stage using the same commercially available software for segmentation and feature extraction: TexRad. While Razik et al. investigated only the discrimination between NMBIC and MBIC [26], Lim et al. also included the distinction between T2 and T3 tumors [17]. The latter paper is the only one which extracted texture features from postoperative images [17]. Razik et al. reported that mean and MPP derived from ADC unfiltered maps showed AUC > 0.8 for the prediction of muscle invasion; however, this resulted from the confounding effect of high-grade tumor histology [26]. Lim et al. concluded that entropy of primary bladder tumors extracted from ADC maps with a 6 mm spatial scale filter can differentiate between <=T2 and >=T3 categories (AUC = 0.85), and between T1 and >=T2 categories (AUC = 0.76) [17].

Among radiomics scores, those developed by Hammouda et al. [24] and Xu et al. [16] achieved the best performance of muscle invasion prediction, with AUCs of 0.98. These models consist of histogram and texture features derived from T2WI and DWI [16] and T2WI, DWI, and ADC, respectively [24]. However, none of these results were validated in a separate cohort. Among papers which included validation sets, Liu et al. reported the highest AUC, with a value of 0.962 in the test set and 0.907 in the validation set [31]. Their radiomics score was the only one that included features extracted from tri-parametric MRI, including T2WI, DWI, ADC, and DCE sequences.

In two papers [24,36], the authors also applied deep learning techniques for predicting tumor stage. The CAD (computer-aided diagnosis) system proposed by Hammouda et al. using neural networks as an ML-classifier performed better than DL methods (AUC of CAD system = 0.986 versus AUCs of DL approaches: 0.796 and 0.743, respectively) [24]. In contrast, in the paper of Li et al., the multi-task DL method exhibited a higher diagnostic performance (AUC = 0.932) compared to the SVM-based radiomics model (AUC = 0.920) [36]. 

Five studies combined radiomics signatures with semantical features to construct complex models [19,21,22,28,32]. VI-RADS score was a parameter included in two of these models [28,32]. In four papers, the complex models performed better than the Rad-Score, achieving AUCs up to 0.970 and 0.943 in the training and validation sets, respectively [28].

Our literature search revealed only one publication which aimed to predict the lymph node (LN) status based on radiomics features extracted from the primary tumor [15]. Their proposed Rad-Score achieved an optimism-corrected AUC of 0.887 in the training set, which was confirmed in the validation set with an AUC of 0.8447. By combining the Rad-Score with the MRI-reported lymph node status, the researchers constructed a complex model which predicted the LN status with AUCs of 0.9118 and 0.8902 in the training and validation sets, respectively.

### 3.5. Prediction of BCa Prognosis

Four studies explored the effectiveness of MRI-based radiomics features for the prediction of BCa prognosis [20,23,25,33]. The papers of Zhang et al. [33] and Kimura et al. [25] focused on the prediction of tumor response to neoadjuvant therapy. Kimura et al. demonstrated that texture features derived from an ADC map can predict the chemoradiotherapy sensitivity of BCa, building a model based on ADC texture features which achieved AUC = 0.96 [25]. Zhang et al. investigated only the response to neoadjuvant chemotherapy (NAC) [33]. They developed a radiomics model with features extracted from T2WI, DWI, and ADC images which exhibited a better performance than single-modality models, yielding an AUC = 0. 96. Moreover, their proposed nomogram which combined Rad-Score with the clinical T stage achieved AUC = 0.973. 

Progression-free survival and 2-year recurrence risk were the outcomes evaluated by the publications of Zhang et al. [23] and Xu et al. [20]. In the first study, the authors extracted features only from DWI images and constructed a signature which achieved a moderate C-index in both the training and validation groups (C-index: 0.640 and 0.612, respectively) [23]. However, their combined nomogram including radiomics signature, age, sex, NAC status (yes or no), RC status (yes or no), Ki-67, presence of carcinoma in situ, and clinical T and N stages, was significantly better compared to radiomics signature alone, with C-indexes of 0.739 and 0.702. Xu et al. created a multiparametric radiomics signature which showed good performance in the training and validation sets (AUCs = 0.85 and 0.82) [20]. By also including the muscle-invasive status, their combined nomogram outperformed the Rad-Score for the first 2-year recurrence risk stratification, with AUCs of 0.915 and 0.838. The reported C-indexes for patients’ recurrence-free survival estimation were 0.832 (radiomics model) and 0.897 (combined model), evaluated only in the training cohort. 

## 4. Discussion

This present systematic review provides an overview of the studies published on the subject of MRI-based radiomics in relation to bladder cancer. All enrolled papers have been published within the last 6 years, reinforcing the idea that radiomics is a recently emerging domain. To the best of our knowledge, there are only a few reviews that aimed to explore the use of radiomics in bladder cancer [38,39,40,41]. However, they included papers which extracted features from both MRI and CT images, and their interest was on studies which evaluated the discrimination between NMBIC and MBIC. 

We chose to include only studies that investigated the use of MRI-based radiomics features since multiparametric MRI is the imaging modality of choice regarding primary and recurrent bladder tumor detection, local staging, and assessment of treatment response [42]. Also, we selected all publications that use MRI-based radiomics features, regardless of their purpose, in order to provide a thorough description of the current state of evidence in this area of research.

We evaluated the quality of enrolled studies using RQS and QUADAS tools. The average RQS score was 11.7, ranging from 3 to 19. Only four papers achieved a RQS equal to or greater than 50% [21,22,28,35].

The majority of papers (*n* = 21) provided detailed information about the imaging protocol, type of scanner, and MRI sequences used for feature extraction. Over two-thirds of studies (*n* = 20) chose a volumetric approach for image segmentation, thus extracting features from the entire tumor volume. Manual or semi-automated image segmentation (usually with manual correction) were the most often encountered methods, with only one paper using deep learning-based image segmentation [24]. In 23 articles, multiple segmentation was performed by two or three radiologists. However, the intraclass correlation coefficient was employed only in 14 studies [15,19,21,23,26,27,28,29,30,32,33,34,35,37] in order to select the most robust radiomics features, while in the remaining articles, a consensus between radiologists was made regarding the final segmented volume. 

After segmentation, the next step in the radiomics workflow is image processing. The RQS does not make any reference to it (except the use of phantom studies), even though the settings used in image processing significantly influence the robustness of radiomic features [43,44]. This essential step aims to standardize the images before extracting radiomic features in terms of factors such as pixel spacing, grey-level intensities, and the grey-level histogram bins. From the papers selected for this review, 12 offered information about image preprocessing [12,14,15,16,18,21,22,24,25,29,29,34], and data normalization methods were reported in 15 articles [12,13,14,16,19,21,27,28,28,29,31,32,33,34,37].

Regarding feature extraction, most of the studies extracted both first- and second-order texture features, and 14 papers analyzed original and filtered images [15,17,18,21,23,26,28,29,31,32,33,34,35,37]. Feature reduction and discrimination statistics were commonly employed for the construction and evaluation of the developed radiomics models, being reported in almost all studies. Features derived from filtered images (mainly Laplacian of Gaussian (LoG) and wavelet transformation) were most frequently included in the optimal subset of parameters in the radiomics signatures. The LoG filter involves a two-step process. First, the image is subjected to Gaussian filtering to reduce noise and smooth the image. Second, Laplacian filtering is applied to identify edges within the image. The width of the Gaussian kernel is controlled by a parameter, which can be adjusted to highlight either finer (small) or coarser (large) textures. The wavelet transformation produces eight decompositions per image by applying either a high or a low pass filter in each of the three dimensions. The resulting filtered features are high-dimensional radiomics features which offer more insights into tumor biological behavior and heterogeneity compared to low-level radiomics features [45,46,47]. 

The proposed radiomics models mainly consisted of second-order features derived from Gray-Level Co-occurrence Matrix (GLCM) and Gray-Level Size Zone Matrix (GLSZM). GLCM-based features, proposed by Haralick et al. [48], describe the spatial relationships of pairs of pixels by calculating the correlation between two gray levels with certain directions and distances. GLSZM-based features, described by Thibault et al. [49], describe the amount of homogeneous connected areas within the region of interest (ROI), of a certain size and intensity. Therefore, these two categories of features can be used for quantitatively assessing the heterogeneity within a segmented region. 

Apart from evaluating only the role of radiomics features, 9 papers also conducted multivariable analysis with non-radiomics features in order to provide a more holistic model [19,20,21,22,27,28,32,33,35]. In all cases, the complex radiomic–clinical signatures outperformed the single-radiomics ones. Cut-off analysis was performed in 8 papers [15,20,21,22,23,26,28,29]. However, feature-by-feature cut-off analysis is not mandatory for studies using machine learning techniques since this might increase the complexity and reduce the interpretability of the final model [50]. 

Almost all studies compared their results with gold standards such as histopathological grade, T stage, or N stage. Additionally, most papers assessed the correlation between radiomics and biological features, mostly focusing on tumor grade or muscle invasion. Only the investigations which evaluated radiomics for the prediction of tumor prognosis did not include these two criteria of RQS [20,23,25,33]. 

The use of calibration statistics and decision curve analysis were present in just over one-third of the studies (*n* = 9) [15,20,21,22,23,28,29,33,35]. Decision curve analysis is an important key of the RQS since it addresses the current and potential application of the developed model in a clinical setting. 

None of the studies performed phantom studies, imaging at multiple points, or cost-effectiveness analyses. Moreover, all of them had a retrospective design, therefore losing 7 important points from the total RQS. 

The lack of open data or codes was another common drawback of the included papers. There were only three studies that used either open-source code, open-source images, or published the calculated set of radiomics features [16,21,35]. In the paper by Xu et al. [16], the authors provided the developed code for feature extraction by uploading it on GitHub for further application. Zheng et al. tested the performance of their radiomics signature and radiomic–clinical nomogram on 13 patients with BCa using data from the open-access database of The Cancer Imaging Archive [21]. By applying the cutoff value defined in the training set, their radiomics score correctly identified 11 out of 13 tumors from the TCIA database as high-risk for muscular invasion. Li et al. published the set of extracted features included in the Rad-Score for each patient (anonymously) [35]. Sharing code and data through “Open Science” is an important step for increasing the reproducibility of subsequent studies. It can also contribute to building larger public databases, which can enhance radiomic studies in the future.

Sixteen out of twenty-six studies performed validation of their radiomics models. However, most of them validated their signatures internally by splitting the data into two sets: training and validation. Only four studies performed external validation from another center [21,22,32,36], reporting promising results. While internal validation is an important step, it should be considered as a preliminary evaluation, and it can overestimate the performance of the model [51]. Therefore, it is crucial to perform external validation to confirm the generalizability of the models. 

Our review has some limitations. First, due to the heterogeneity between the included studies, we could not carry out a meta-analysis. The studies differed in terms of image preprocessing, applied filters, feature extraction, and machine-learning algorithms used. Second, we included only studies that developed radiomics models using ML methods. We chose not to perform RQS analysis on papers that employed deep learning since they would have not met some criteria of the score, thus leading to an unfair comparison. Also, RQS is a relatively new tool that has its own limitations and requires further improvements. Last but not least, we cannot ignore a possible publication bias since none of the included papers reported negative results.

## 5. Conclusions

In conclusion, this systematic review has demonstrated the potential of radiomics as a promising tool for the personalized management of patients with BCa. In order to fully leverage its benefits and translate radiomics into clinical practice, future studies should aim for standardized radiomics workflow, open-source data, prospective investigations, and external validation. 

## Figures and Tables

**Table 1:** General characteristics of included studies.

Author (Year of Publication)	Journal	Study Design	No of Patients (Train vs. Test Cohort)	Surgical Technique	Reference Standard	Analyzed Outcome	MRI Sequence	Readers	Imaging Timing	Provided Protocol	Scanner
Xu et al. (2017) [12]	Abdominal Radiology	Retrospective	68	TURBT	Pathological T stage	Muscle invasion	T2WI	2	Prior to TURBT	yes	GE Discovery 750 3.0T
Zhang et al. (2017) [13]	Journal of Magnetic Resonance Imaging	Retrospective	61	NA	Pathological grade	Tumor grade	DWI and ADC	2	prior to treatment	yes	GE Discovery 750 3.0T
Tong et al. (2018) [14]	Advances in Radiation Oncology	Retrospective	65	RC	Pathological T stage	Muscle invasion	T2WI	2	Prior/after treatment	yes	1.5 and 3.0T scanners
Wu et al. (2018) [15]	EBioMedicine	Retrospective	103 (69:34)	RC	Pathological N stage	Lymph node status	T2WI	2	Preoperative	yes	Philips Intera Achieva 3.0T
Xu et al. (2019) [16]	Journal of Magnetic Resonance Imaging	Retrospective	54	NA	Pathological T stage	Muscle invasion	T2WI, DWI and ADC	3	Preoperative	yes	GE Discovery 750 3.0T
Lim et al. (2019) [17]	American Journal of Roentgenology	Retrospective	36	TURBT and RC	Pathological T stage	Tumor stage (muscle invasion and extravesical disease)	T2WI and ADC	2	Post TURBT, prior to RC	yes	1.5 and 3.0T scanners
Wang et al. (2019) [18]	European Radiology	Retrospective	100 (70:30)	TURBT or RC	Pathological grade	Tumor grade	T2WI, DWI and ADC	2	NA	yes	Siemens Magnetom Trio, 3.0T
Xu et al. (2020) [19]	European Radiology	Retrospective	218 (131:87)	TURBT and RC	Pathological T stage	Muscle invasion	DWI and ADC	2	Prior to TURBT	yes	Philips Ingenia 3.0T MR
Xu et al. (2019) [20]	Journal of Magnetic Resonance Imaging	Retrospective	71 (50:21)	TURBT or RC	NA	Recurrence Risk	T2WI, DWI, DCE	2	Preoperative	yes	Siemens Magnetom 3.0T MR
Zheng et al. (2019) [21]	Cancer	Retrospective	199 (130:69)	TURBT or RC	Pathological T stage	Muscle invasion	T2WI	2	Prior to treatment	yes	Philips Achieva 3.0T
Wang et al. (2020) [22]	European Radiology	Retrospective	106 (64:42)	RC or PC or TURBT	Pathological T stage	Muscle invasion	T2WI, DWI and ADC	3	Preoperative	yes	Siemens Magnetom 3.0T/GE Discovery 750 3.0T
Zhang et al. (2020) [23]	European Journal of Radiology	Retrospective	210 (105:105)	TURBT or RC or CT or RT	NA	Progression-free Survival	DWI	2	NA	yes	Philips Ingenia 3.0T MR scanner
Hammouda et al. (2021) [24]	Computerized Medical Imaging and Graphics	Retrospective	42	NA	Pathological T stage	Muscle invasion	T2WI, DWI, ADC	NA	NA	yes	Philips Ingenia 3.0T
Kimura et al. (2022) [25]	EuropeanRadiology	Retrospective	45	PC or RC	Pathological T stage	Response to NCT	ADC	2	Prior to treatment	yes	Philips Intera Achieva 1.5T
Razik et al. (2021) [26]	The British Journal of Radiology	Retrospective	40	NA	Pathological grade	Muscle invasion + grade	T2WI, DWI and ADC	2	prior to treatment	yes	Philips Achieva 3.0T
Zheng et al. (2021) [27]	Abdominal Radiology	Retrospective	294	TURBT or RC	Pathological grade	Tumor grade	T2WI, DCE	2	Preoperative	yes	Siemens Magnetom Verio 3.0T
Zheng et al. (2021) [28]	Frontiers in Oncology	Retrospective	185 (129:56)	NA	Pathological T stage	MIBC	T2WI and DCE	2	Preoperative	yes	Siemens Magnetom Verio 3.0T
Zheng et al. (2021) [29]	Cancer Imaging	Retrospective	179 (125:54)	TURBT or RC	Immunohistochemistry	Ki-67	T2WI and DCE	2	Preoperative	yes	Siemens Magnetom Verio 3.0T
Feng et al. (2022) [30]	Life	Retrospective	74 (58:16)	RC or PC or TURBT	Pathological grade	Tumor grade	ADC 1000, ADC 1700, ADC 3000	2	prior to treatment	yes	GE Discovery 750 3.0T
Liu et al. (2023) [31]	Academic Radiology	Retrospective	206 (165:41)	NA	Pathological T stage	Muscle invasion	T2WI, DWI, DCE	3	prior to treatment	yes	Siemens Magnetom Trio 3.0T
Wang et al. (2022) [32]	Urologic Oncology	Retrospective	191 (121:70)	TURBT or RC	Pathological T stage	Muscle invasion	DWI	2	Preoperative	yes	GE Discovery 750 3.0T/United Imaging uMR790 3.0T
Zhang et al. (2022) [33]	Frontiers in Oncology	Retrospective	70	TURBT or RC or PC	Pathological T stage	Response to chemotherapy	T2, DWI, ADC	2	Prior to treatment	yes	GE Discovery 750 3.0T
Zheng et al. (2022) [34]	Cancers	Retrospective	111 (77:34)	NA	Immunohistochemistry	CD8A	T2WI + DCE	2	Preoperative	yes	Siemens Magnetom 3.0T MR
Li et al. (2023) [35]	Frontiers in Oncology	Retrospective	169 (118:51)	NA	Pathological grade	Tumor grade	T2WI and ADC	2	prior to treatment	yes	Philips Ingenia and Ingenia X 3.0T MR
Li et al. (2023) [36]	Computer Methods and Programs in Biomedicine	Retrospective	121 (93:28)	TURBT or RC or PC	Pathological T stage	Muscle invasion	T2WI	1	Preoperative	yes	Siemens Magnetom Skyra 3.0T/United Imaging Healthcare 3.0T
Liu et al. (2023) [37]	Bioengineering	Retrospective	111 (77:34)	NA	RNA sequencing	Immune Prognostic Signature	T2WI + DCE	2	Preoperative	yes	Siemens Magnetom 3.0T

NA = not available, RC = radical cystectomy, PC = partial cystectomy, DCE = dynamic contrast enhanced images, CT = chemotherapy.

**Table 2:** RQS results.

Reference	1. Image Protocol Quality	2. Multiple Segmentations	3. Phantom Study	4. Imaging at Multiple Time Points	5. Feature Reduction/Adjustment for Multiple Testing	6. Multivariable Analysis with Non-Radiomics Features	7. Biological Correlates	8. Cut-Off Analysis	9. Discrimination Statistics	10. Calibration Statistics	11. Prospective Study	12. Validation	13. Comparison to “Gold standard”	14. Potential Clinical Utility	15. Cost-Effectiveness Analysis	16. Open Science and Data	Total	RQS
Score range	0–2	0–1	0–1	0–1	−3–3	0–1	0–1	0–1	0–2	0–2	0–7	−5–5	0–2	0–2	0–1	0–4	−8–36	0–100%
Xu et al. (2017) [12]	1	0	0	0	3	0	1	0	2	0	0	−5	2	0	0	0	4	11%
Zhang et al. (2017) [13]	1	1	0	0	3	0	1	0	2	0	0	−5	2	0	0	0	5	14%
Tong et al. (2018) [14]	1	1	0	0	3	0	1	0	2	0	0	2	2	0	0	0	12	33%
Wu et al. (2018) [15]	1	1	0	0	3	1	1	1	2	1	0	2	2	2	0	0	17	47%
Xu et al. (2019) [16]	1	1	0	0	3	0	1	0	2	0	0	−5	2	0	0	1	6	17%
Lim et al. (2019) [17]	1	0	0	0	3	0	1	0	1	0	0	−5	2	0	0	0	3	8%
Wang et al. (2019) [18]	1	1	0	0	3	0	1	0	2	0	0	2	2	0	0	0	12	33%
Xu et al. (2020) [19]	1	1	0	0	3	1	1	0	1	0	0	2	2	0	0	0	12	33%
Xu et al. (2019) [20]	1	1	0	0	3	1	1	1	2	1	0	2	2	2	0	0	17	47%
Zheng et al. (2019) [21]	1	1	0	0	3	1	1	1	2	1	0	3	2	2	0	1	19	53%
Wang et al. (2020) [22]	1	1	0	0	3	1	1	1	2	1	0	3	2	2	0	0	18	50%
Zhang et al. (2020) [23]	1	1	0	0	3	1	0	1	2	1	0	2	0	2	0	0	14	39%
Hammouda et al. (2021) [24]	1	1	0	0	3	0	1	0	2	0	0	2	2	0	0	0	12	33%
Kimura et al. (2022) [25]	1	1	0	0	3	0	1	0	2	0	0	−5	2	0	0	0	5	14%
Razik et al. (2021) [26]	1	1	0	0	3	0	1	1	1	0	0	−5	2	0	0	0	5	14%
Zheng et al. (2021) [27]	1	1	0	0	3	1	1	0	2	1	0	2	2	2	0	0	16	44%
Zheng et al. (2021) [28]	1	1	0	0	3	1	1	1	2	1	0	2	2	2	1	0	18	50%
Zheng et al. (2021) [29]	1	1	0	0	3	0	1	1	2	1	0	2	0	2	0	0	14	39%
Feng et al. (2022) [30]	1	0	0	0	3	0	1	0	2	0	0	2	2	0	0	0	11	31%
Liu et al. (2023) [31]	1	1	0	0	3	0	1	0	2	0	0	2	2	0	0	0	12	33%
Wang et al. (2022) [32]	1	1	0	0	3	1	1	0	1	0	0	3	2	0	0	0	13	36%
Zhang et al. (2022) [33]	1	1	0	0	3	1	1	0	1	0	0	−5	2	2	0	0	7	19%
Zheng et al. (2022) [34]	1	1	0	0	3	0	1	0	2	0	0	2	0	0	0	0	10	28%
Li et al. (2023) [35]	1	1	0	0	3	1	1	0	2	1	0	2	2	2	0	1	19	53%
Li et al. (2023) [36]	1	1	0	0	3	0	1	0	2	0	0	3	2	0	0	0	13	36%
Liu et al. (2023) [37]	1	1	0	0	3	0	1	0	2	0	0	2	0	0	0	0	10	28%

**Table 3:** QUADAS-2 results (Green = Low risk of bias, Red = High risk of bias, Orange = Unclear risk of bias).

Study	Risk of Bias				Applicability Concerns		
	Patient Selection	Index Test	Reference standard	Flow & Timing	Patient Selection	Index Test	Reference standard
Xu et al. (2017) [12]	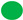	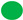	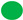	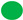	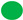	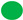	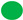
Zhang et al. (2017) [13]	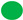	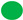	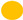	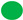	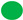	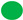	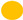
Tong et al. (2018) [14]	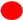	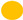	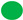	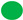	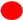	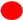	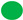
Wu et al. (2018) [15]	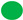	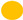	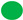	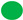	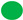	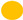	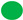
Xu et al. (2019) [16]	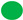	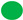	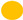	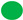	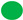	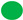	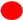
Lim et al. (2019) [17]	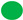	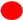	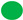	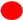	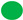	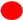	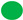
Wang et al. (2019) [18]	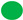	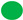	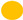	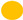	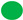	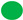	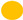
Xu et al. (2020) [19]	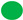	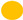	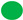	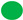	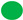	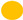	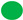
Xu et al. (2019) [20]	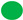	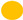	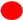	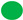	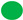	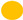	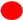
Zheng et al. (2019) [21]	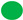	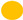	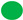	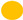	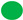	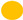	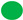
Wang et al. (2020) [22]	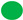	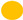	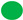	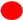	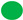	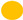	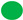
Zhang et al. (2020) [23]	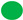	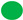	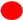	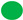	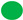	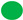	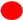
Hammouda et al. (2021) [24]	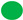	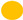	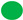	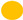	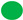	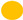	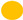
Kimura et al. (2022) [25]	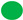	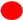	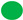	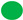	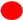	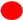	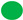
Razik et al. (2021) [26]	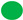	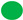	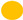	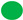	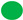	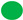	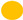
Zheng et al. (2021) [27]	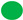	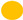	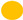	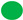	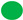	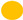	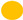
Zheng et al. (2021) [28]	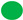	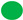	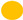	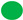	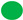	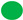	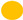
Zheng et al. (2021) [29]	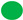	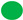	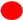	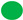	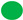	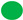	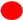
Feng et al. (2022) [30]	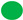	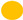	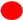	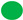	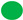	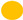	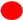
Liu et al. (2023) [31]	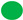	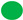	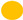	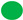	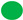	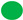	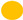
Wang et al. (2022) [32]	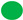	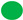	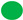	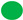	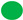	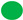	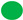
Zhang et al. (2022) [33]	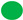	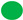	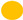	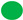	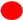	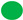	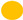
Zheng et al. (2022) [34]	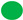	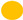	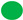	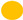	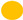	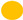	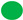
Li et al. (2023) [35]	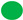	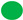	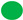	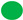	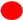	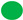	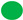
Li et al. (2023) [36]	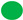	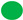	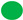	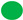	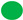	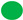	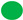
Liu et al. (2023) [37]	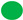	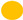	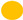	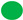	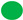	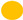	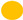

## Data Availability

The data presented in this study are available in this article, including Supplementary Files.

## Supplementary Materials

The following supporting information can be downloaded at: https://www.mdpi.com/article/10.3390/diagnostics13132300/s1, Table S1: Characteristics of the radiomics research for prediction of BCa grade and molecular correlates; Table S2: Characteristics of the radiomics research for prediction of Bca stage, including muscle invasion and N stage; Table S3: Characteristics of the radiomics research for prediction of Bca prognosis.
