# Supplementary material for: MRI-Based Radiomics in Bladder Cancer: A Systematic Review and Radiomics Quality Score Assessment
Source: Diagnostics (Basel). 2023 Jul 6;13(13):2300. doi: 10.3390/diagnostics13132300 (PMC10341244; doi:10.3390/diagnostics13132300)
Supplement: Supplementary file 1 [file diagnostics-13-02300-s001.zip › diagnostics-2438183-supplementary.pdf]

Supplementary Files

Table S1. Characteristics of the radiomics research for prediction of BCa grade and molecular correlates.

Author	Data augmentation	Data resampling methods	Segmentation Modality	Segmentation Software	Feature extraction software	Features Category	No of extracted features	ML method for Feature Selection	Radiomic model - best performance		Combined model	
									Relevant radiomics features (number)	AUC Train/Validation	Variables	AUC Train/Validation
Zhang et al [13]	No	10-fold cross-validation	Manual segmentation VOI	MathLab R2012b	NA	histogram + glcm	102	SVM-RFE	22	0.861	NA	NA
Wang et al [18]	No	10-fold cross-validation	Manual segmentation VOI	NA	PyRadiomics	shape, first-order, texture (Original Images, LoG filtered Images, wavelet transformation)	2772	LASSO+ LR	7	0.9233 / 0.9276	NA	NA
Zheng et al [27]	SMOTE	10-fold cross-validation	Manual segmentation VOI	ITK-SNAP	PyRadiomics	shape, first-order, texture (Original Images, LoG filtered Images, wavelet transformation)	2436	SVM-RFE, LASSO (best), RFS-FS	26	0.961 / 0.952	Radiomics score + VI-RADS score	0.956 / 0.958
Feng et al [30]	No	10-fold cross-validation	Manual segmentation VOI	ITK-SNAP	LIFEx	shape + statistical indexes + first-order + texture	147	LASSO + LR	7	0.901 vs 0.920	NA	NA
Razik et al [26]	No	NA	Manual segmentation ROI	TexRAD	TexRAD	first-order	108	NA	2	0.897	NA	NA
Zheng et al [29]	SMOTE	10-fold cross validation	Manual segmentation, VOI	ITK-SNAP	PyRadiomics	shape, first-order, texture (Original Images, LoG filtered Images, wavelet transformation)	2436	LASSO (best), SVM	9	0.859/0.819		NA

Zheng et al [34]	No	10-fold cross validation	Manual segmentation, VOI	ITK-SNAP	PyRadiomics	shape, first-order, texture (Original Images, LoG filtered Images, wavelet transformation, exponential, logarithm, square, square root and gradient filtered images)	3562	LASSO	9	0.857/0.844	NA	NA
Li et al [35]	No	10-fold cross-validation	Manual segmentation VOI	ITK-SNAP	PyRadiomics	shape+size, first-order, texture, (original images, LoG filtered images, wavelet transformation)	3148	LASSO	24	0.942/0.910	Radiomics score + age + number of tumours	0.955/ 0.931
Liu et al [37]	no	10-fold cross validation	Manual segmentation, VOI	ITK-SNAP	PyRadiomics	shape+size, first-order, texture, (original images, Log filtered images, wavelet transformation)	3562	LASSO	10	0.839/0.810	NA	NA

Table S2: Characteristics of the radiomics research for prediction of BCa stage, including muscle invasion and N stage.

Author	Data augmentation	Data resampling methods	Segmentation Modality	Segmentation Software	Feature extraction software	Features Category	No of extracted features	ML method for Feature Selection	Radiomic model - best performance		Combined model	
									No. Relevant radiomics features (sequence)	AUC Train/ Validation	Variables	AUC Train/Validation
Xu et al [12]	SMOTE	10-fold cross validation	Manual segmentation, VOI	Custom developed Package; MATLAB R2012b	NA	Histogram, ND Haralick	63	SVM-RFE	13 (T2WI)	0.861	NA	NA
Tong et al [14]	no	Cross validation with a leave-one-out method	Manual segmentation, VOI	NA	NA	LBP (local binary pattern),GLCM	15834	SVM	9 (T2WI)	0.806	NA	NA
Wu et al [15]	no	10-fold cross validation	Manual segmentation, VOI	3D Slicer	PyRadiomics	shape, first-order, texture (Original Images, LoG filtered Images, and Wavelet transformation)	718	LASSO	9 (T2WI)	0.900/ 0.847	RadScore + MRI-reported LN status	0.911/ 0.890
Xu et al [16]	SMOTE	10-fold cross validation	Manual segmentation, ROI	custom-developed package	MATLAB R2015b	Histogram,CM, RLM	1104	SVM-RFE	19 (T2WI+DWI+ ADC)	0.985	NA	NA
Lim et al [17]	no	NA	Manual segmentation, ROI	TexRAD	TexRAD	First-order	36	NA	1	T2 vs T3 stage: 0.85 (T2WI), 0.80 (ADC) T1 vs T2 stage: 0.76 (ADC)	NA	NA

Xu et al [19]	NA	NA	Manual segmentation, VOI	ITK-SNAP	MATLAB R2016a	shape, first-order, texture	156	RF (best), AR	73	0.907/ 0.904	RadScore + result of TURBT	0.897
Zheng et al [21]	No	Bootstrapping method	Semiautomatic segmentation, VOI	3D Slicer	PyRadiomics	Shape, first-order, texture (Original Images, LoG filtered Images, and Wavelet transformation)	2602	LASSO	23	0.913/ 0.874	RadScore + MRI-determined tumor size	0.922/ 0.876
Wang et al [22]	NA	10-fold cross validation	Manual segmentation, ROI	Custom developed Package; MATLAB 2016b	MATLAB	Histogram,CM , RLM,NGTDM, GLSZM	1404	SVM-RFE + LR (best) ,LASSO	36 (T2+ADC+ DWI)	0.880/ 0.813	RadScore + tumor stalk	0.924/ 0.877
Hammouda et al [24]	no	Cross validation with a leave-one-out method	Automatic segmentation, VOI	CNN1 and CNN2 based on DeepMedic network	NA	Histogram, GLCM,GLRLM, morphological	157	NN (best), RF, SVM	157	0.986	NA	NA
Razik et al [26]	No	NA	Manual segmentation, ROI	TexRAD	TexRAD	first-order	108	NA	1	0.819	NA	NA
Zheng et al [28]	no	10-fold cross validation	Manual segmentation, VOI	ITK-SNAP	PyRadiomics	shape+size, first-order, texture (Original Images, LoG filtered Images, and Wavelet transformation)	2436	SVM-RFE,LASSO (best), RF	21	0.934/ 0.906	RadScore + VI-RADS score	0.970/ 0.943
Liu et al [31]	no	10-fold cross validation	Manual segmentation, ROI	ITK-SNAP	PyRadiomics	shape+size, first-order, texture (Original Images, LoG filtered Images, wavelet transformation, exponential, logarithm,	4128	LASSO	28	0.962/ 0.907	NA	NA

						square, square root ,gradient filtered images, LBP)						
Wang et al [32]	no	5-fold cross validation	Manual segmentation, VOI	ITK-SNAP	PyRadiomics	shape, first-order, texture, (Original Images, LoG filtered Images, wavelet transformation)	1070	LASSO (best), ExtraTrees, LightGBM, eXtremeGradientBoost, Adaboost, CatBoost, LR, GradientBoosting, SVM, RF, Decision tree	6	0.801/ 0.806	RadScore + VI-RADS	0.889/0.881 (reader1) 0.854/0.844 (reader 2)
Li et al [36]	no	5-fold cross validation	Manual segmentation, VOI	ITK-SNAP	PyRadiomics	shape, first-order, texture, transformation	851	SVM (best), LR, GBDT (gradient boosting decision tree), RF)	48	0.920/ 0.844	NA	NA

Table S3: Characteristics of the radiomics research for prediction of BCa prognosis

Author	Data augmentation	Data resampling methods	Segmentation Modality	Segmentation Software	Feature extraction software	Features Category	No of extracted features	ML method for Feature Selection	Radiomic model - best performance			Combined model		
									Relevant radiomics features (number)	AUC Train/ Validation	C index	Variables	AUC Train/ Validation	C index
Xu et al [20]	no	5-fold cross validation	Manual segmentation, ROI	custom-developed package	MATLAB R2015b	histogram, CM, RLM, NGTDM, GLSZM	1872	SVM-RFE (best), LASSO	32	0.8593/ 0.8219	0.832/ 0.897	RadScore + muscle-invasive state	0.915/ 0.838	
Zhang et al [23]	no	10-fold cross validation	Manual segmentation, VOI	ITK-SNAP	PyRadiomics	shape, first-order, texture (Original Images, LoG filtered Images, wavelet transformation)	2250	LASSO	8	NA	0.640/ 0.612	NA	NA	0.739/ 0.702
Kimura et al [25]	no	10-fold cross validation	Manual segmentation, VOI	LifeX	LifeX	statistical, first-order histogram, shape, GLCM, GLRLM, NGLDM, GLZLM	46	RF, SVM (best)	3	0.96	NA	NA	NA	NA
Zhang et al [33]	no	NA	Manual segmentation, VOI	ITK-SNAP	PyRadiomics	shape, first-order, GLCM, GLRLM, GLSZM, GLDM, NGTDM (Original Images, LoG filtered Images, wavelet transformation), LBP	1316	LR	9	0.967	NA	RadScore + clinical T stage	0.973	NA
